# Interplay of DNA methyltransferase 1 and EZH2 through inactivation of Stat3 contributes to β-elemene-inhibited growth of nasopharyngeal carcinoma cells

**DOI:** 10.1038/s41598-017-00626-6

**Published:** 2017-03-30

**Authors:** JingJing Wu, Qing Tang, LiJuan Yang, YuQing Chen, Fang Zheng, Swei Sunny Hann

**Affiliations:** Laboratory of Tumor Biology, Guangdong Provincial Hospital of Chinese Medicine, The Second Clinical Medical Collage, Guangzhou University of Chinese Medicine, Guangzhou, Guangdong Province 510120 China

## Abstract

β-elemene, a compound extracted from Curcuma wenyujin plant, exhibits anticancer activity in many cancer types. However, the detailed mechanism by which β-elemene inhibits growth of nasopharyngeal carcinoma (NPC) cells remains unknown. We showed that β-elemene reduced phosphorylation of signal transducer and activator of transcription 3 (Stat3), and protein expressions of DNA methyltransferase 1 (DNMT1) and enhancer of zeste homolog 2 (EZH2). Exogenously expressed Stat3 antagonized the effect of β-elemene on DNMT1 and EZH2 expressions. Furthermore, overexpressions of DNMT1 and EZH2 reversed the effect of β-elemene on phosphorylation of Stat3 and cell growth inhibition. Intriguingly, exogenously expressed DNMT1 overcame β-elemene-inhibited EZH2 protein expression and promoter activity. On the contrary, silencing of EZH2 and DNMT1 genes feedback strengthened the effect of β-elemene on phosphorylation of Stat3. Consistent with this, β-elemene inhibited tumor growth, phosphorylation of Stat3, expressions of DNMT1 and EZH2 in a mouse xenograft model. Collectively, this study shows that β-elemene inhibits NPC cell growth via inactivation of Stat3, and reduces DNMT1 and EZH2 expressions. The interplay of DNMT1 and EZH2, and the mutual regulations among Stat3, EZH2 and DNMT1 contribute to the overall responses of β-elemene. This study uncovers a novel mechanism by which β-elemene inhibits growth of NPC cells.

## Introduction

Human nasopharyngeal carcinoma (NPC) is a squamous cell malignant tumor prominently in Southeast Asia and Southern China. Genetic predisposition, and epigenetic variations, exposure to chemical carcinogens and latent Epstein-Barr virus infection, among others, play important roles in the development of this malignancy^1–4^. Although local radiation and surgery provide good control of NPC, the prognosis of patients with NPC still remains poor due to the advanced stage at the time of diagnosis, regional relapse, and distant metastasis. In addition, the high radiotherapy resistance is a severe obstacle for the treatment of NPC^5, 6^. Moreover, adverse effects, including upper gastrointestinal impairment and bone marrow suppression, depressed the toleration and limited the clinical use of concurrent chemo-radiotherapies. This led us to explore new strategies based on molecular mechanisms and the disease characteristics to improve the therapeutics of patients with NPC.

β-elemene (1-methyl-1-vinyl-2, 4-diisopropenyl-cyclohexane), a naturally occurring compound extracted from the traditional Chinese medicinal herb Zedoary, has been shown to inhibit various cancer types through regulating multiple signaling pathways and targeting genes or/and proteins without severe adverse effects^7–10^. In addition, β-elemene has been shown to reverse the drug resistance and to enhance chemotherapeutic sensitivity in several cancer cells^11–13^. However, the underlying mechanisms associated with its therapeutic efficacy in inhibiting cancer cell growth remain unclear. More importantly, no published data so far have showed the therapeutic potential of β-elemene in the treatment NPC.

DNA methylation plays an essential role in regulating many cellular processes. Aberrant DNA methylation resulted in epigenetic silencing and/or altered gene expressions that contribute to tumor cell invasion and progression. Three active mammalian DNA methyltransferases (DNMT), such as DNMT1, DNMT3a, and DNMT3b, have been identified. Among these, DNMT1 is a major mediator and plays a critical role for maintaining methylation during DNA replication^14^. In addition, DNMT1 also involves in various biological functions, including tumor growth and progression^15–17^. Several lines of evidence demonstrated that high expression of DNMT1 existed in several cancer types including NPC and that targeting DNMT1 suppressed cancer cell growth^17–22^. Thus, inhibition of DNMT1 could be a promising therapeutic potential for treating cancers including NPC.

The enhancer of zeste homolog 2 (EZH2), a polycomb histone methyltransferase, have been shown to play an important role in tumorigenesis and cancer development through epigenetic gene silencing and genetic regulation^22, 23^. EZH2 is highly expressed in several cancer types including NPC and associated with the expression of several target genes involving in growth, metastasis and prognosis of cancers^23–26^. Reports showed that EZH2 inhibitors, such as suberoylanilide hydroxamic acid (SAHA) and 3-deazaneplanocin A (DZNep), exerted anticancer effects through activation of tumor-suppressor microRNAs (miRNAs) in gastric and liver cancer cells^27^. EZH2 contributes to tumor development and progression, and represents an independent prognostic marker in patients with NPC^24^. Thus, targeting EZH2 may be considered as an additional therapeutic potential for the treatment and prevention of NPC.

Signal transducer and activator of transcription factors (Stats) have been shown to regulate several target genes required for tumor cell proliferation and invasion^28^. Accumulated evidence showed that activation and highly expression of Stat3 are found in many cancer types including NPC, and implicate in the development and progression of various tumors suggesting the most promising new target for cancer therapy^29, 30^. Long-palate, lung and nasal epithelium clone 1 (LPLUNC1), a promising candidate tumor suppressor gene was associated with tumorigenesis of NPC; LPLUNC1 inhibited proliferation and promoted apoptosis by suppressing the Stat3 pathway in NPC cells^31^. Together, these findings implied that blockade of Stat3 could also be an additional therapeutic strategy for NPC.

The links of EZH2 and DNMT1, the two epigenetic regulators and oncogenes, have been shown to be associated with tumorigenesis and cancer progression in several other studies^32–34^. EZH2- and DNMT1-mediated epigenetic regulation contributed to the growth and progression of different cancer cells^35^. In addition, early studies found that the DNMT1 and EZH2 gene promoters contained putative Stat3 binding sites and that regulation of Stat3 signaling altered the expression of DNMT1, EZH2, and downstream signaling^36, 37^. Nevertheless, the detailed mechanisms underlying the regulation of these factors in converging on the occurrence and progression of NPC remain to be determined.

In this study, we explored the potential molecular mechanism underlying the anti-NPC effects by β-elemene.

## Results

### β-elemene inhibited growth and induced cell cycle arrest in NPC cells

**Figure 1:**
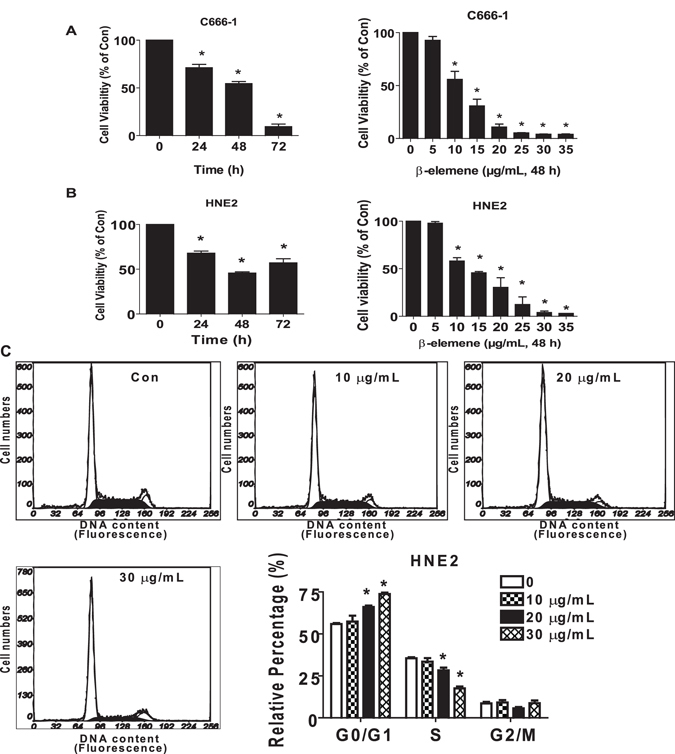
β-elemene inhibited growth and induced cell cycle arrest in NPC cells. (**A,B**) C666-1 (**A**) and HNE2 (**B**) cells were treated with increased concentrations of β-elemene for up to 48 h or to β-elemene (20 μg/mL) for up to 72 h. (**C**) HNE2 cells were treated with indicated concentrations of β-elemene for 24 h. The cells were collected and processed for analysis of cell cycle distribution. Cell cycle was analyzed by flow cytometry after propidium iodide (PI) staining, and the percentages of the cell population in each phase (G0/G1, S and G2/M) of cell cycle were analyzed by Multicycle AV DNA Analysis Software. Data are expressed as a percentage of total cells. Values are given as the mean ± SD, from 3 independent experiments performed in triplicate. *Indicates significant difference as compared to the untreated control group (P < 0.05).

We first test the effect of β-elemene on viability of NPC cells. As shown in Fig. 1A,B, β-elemene inhibited cell viability in the time- and dose-dependent manner with optimal dose of 15 to 25 μg/mL for up to 72 h in C666-1 and HNE2 cells. The IC50 were 10.98 and 12.92 μg/mL in C666-1 and HNE2 cells, respectively. We also performed the cell cycle distribution experiment. As expected, we found that, compared with the untreated control cells, β-elemene at 20 to 30 μg/mL significantly increased the proportion of cells at G0/G1 phases, while the proportion of cells at S phase was reduced (Fig. 1C), suggesting that β-elemene induced cell cycle arrest in G0/G1 phases in HNE2 cells.

### β-elemene reduced the phosphorylation of Stat3

**Figure 2:**
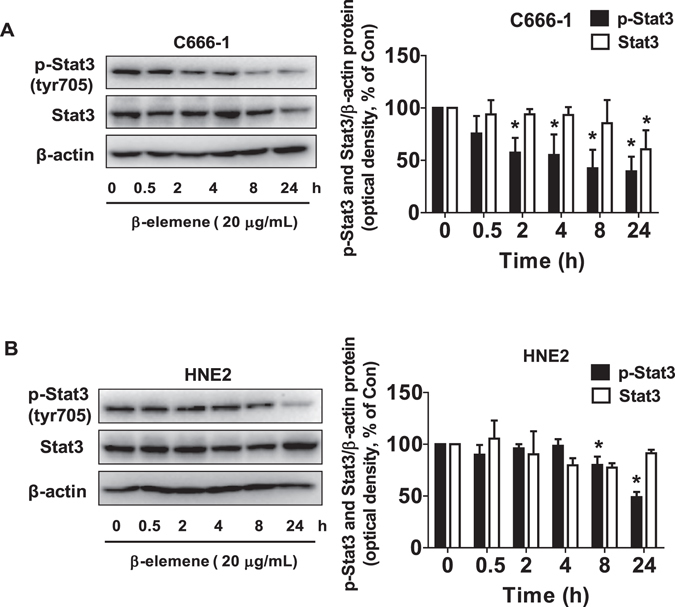
β-elemene reduced the phosphorylation of Stat3. (**A,B**) C666-1 (**A**) and HNE2 (**B**) cells were treated with β-elemene (20 μg/mL) in the indicated times, and cell lysate was harvested and the expression of the phosphorylated or total protein of Stat3 was measured by Western blot analysis using corresponding antibodies. β-actin was used as loading control. The figures are representative cropped gels/blots that have been run under the same experimental conditions. The bar graphs represented the densitometry results of p-Stat3, Stat3/β-actin as mean ± SD of at least three separate experiments. *Indicates significant difference from the untreated control cells (P < 0.05).

Transcription factor Stat3 has been involved in the tumor cell growth and progression^28^. Because of this, we next tested the effect of β-element on Stat3 signaling. We showed that β-elemene reduced the phosphorylation of Stat3 in the time-dependent fashion with significant effects observed at 0.5 h for up to 24 h in C666-1 and HNE2 cells (Fig. 2A,B). Note that β-elemene had little effect on total Stat3 protein expression but inhibited at 24 h in C666-1 cells (Fig. 2A,B).

### β-elemene inhibited the protein expression of DNMT1 and EZH2 through inactivation of Stat3

**Figure 3:**
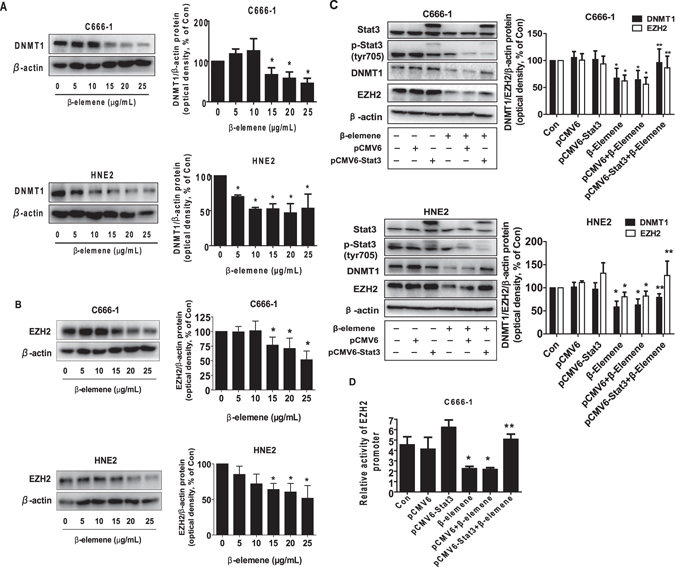
β-elemene inhibited the protein expression of DNMT1 and EZH2 through Stat3. (**A,B**) C666-1 and HNE2 cells were exposed to increased concentrations of β-elemene for 24 h, followed by measuring DNMT1 and EZH2 proteins by Western blot. (**C**) C666-1 and HNE2 cells were transfected with control or Stat3 expression vector described in the Materials and Methods section for 24 h followed by exposure the cells to β-elemene (20 μg/mL) for an additional 24 h. Afterwards, Stat3, p-Stat3, DNMT1 and EZH2 protein expressions were determined by Western blot. (**D**) C666-1 cells were transfected with control or Stat3 expression vector, and with a wild type human EZH2 promoter reporter construct ligated to luciferase reporter gene and internal control secreted alkaline phosphatase for 24 h, followed by treating with β-elemene (20 μg/mL) for an additional 24 h. Afterwards, the promoter activities were determined using the Secrete-Pair Dual Luminescence Assay Kit as described in the Materials and Methods section. Values in bar graphs were given as the mean ± SD from three independent experiments performed in triplicate. *Indicates significant difference as compared to the untreated control group (P < 0.05). **Indicates significant difference from the β-elemene treated alone (P < 0.05).

Studies showed that DNMT1, the major enzyme responsible for maintenance of the DNA methylation pattern, and EZH2, the catalytic subunit of polycomb repressive complex 2 (PRC2), were highly expressed in a wide variety of cancer types^27, 38^. Herein, we examined the potential role of these two factors in mediating the effect of β-elemene on cell growth. We found that β-elemene reduced the protein expressions of DNMT1 and EZH2 in a dose-dependent manner, the optimal responses found at 15 to 25 μg/mL in C666-1 and HNE2 cells, respectively (Fig. 3A,B). We next characterized the potential role of Stat3 in mediating the effect of β-elemene on protein expression of DNMT1 and EZH2. Interestingly, exogenously expression of Stat3 transfected into the C666-1 and HNE2 cells antagonized the β-elemene-inhibited protein levels of DNMT1 and EZH2, while β-elemene concomitantly reduced the phosphorylation of Stat3 in C666-1 and HNE2 cells (Fig. 3C). Moreover, we showed that overexpressed Stat3 reversed in part the β-elemene-inhibited EZH2 promoter activity (Fig. 3D). Together, these results confirmed the upstream role of Stat3 for regulation of DNMT1 and EZH2 in mediating the effect of β-elemene on this process.

### Exogenously expression of DNMT1 overcame the effect of β-elemene on EZH2 protein expression and promoter activity

**Figure 4:**
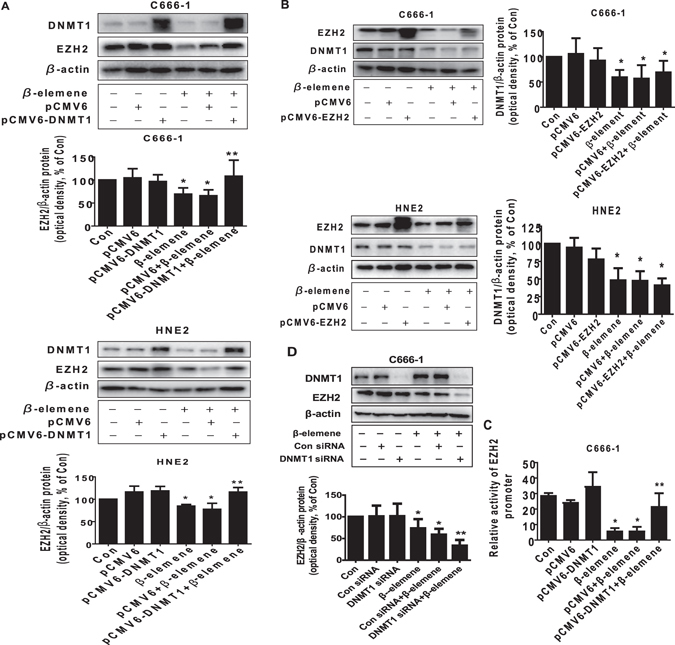
Exogenously expression of DNMT1 overcame the effect of β-elemene on EZH2 protein expression and promoter activity. (**A,B**) C666-1 and HNE2 cells were transfected with control or DNMT1 or EZH2  expression vectors for 24 h prior to exposure of the cells to β-elemene (20 μg/mL) for an additional 24 h. Afterwards, Western blot analysis were used measure the protein levels of DNMT1 and EZH2 using corresponding antibodies. (**C**) C666-1 and HNE2 cells were transfected with control or DNMT1 expression vector, and with a wild type human EZH2 promoter reporter construct ligated to luciferase reporter gene and internal control secreted alkaline phosphatase for 24 h, followed by treating with β-elemene (20 μg/mL) for an additional 24 h. Afterwards, the promoter activities were determined using the Secrete-Pair Dual Luminescence Assay Kit as described in the Materials and Methods section. (**D**) C666-1 cells were transfected with control or DNMT1 siRNA for 24 h prior to exposure of the cells to β-elemene (20 μg/mL) for an additional 24 h. Afterwards, Western blot analysis were used for determining the protein levels of DNMT1 and EZH2 using corresponding antibodies. The figures are representative cropped gels/blots that have been run under the same experimental conditions. Values in bar graphs were given as the mean ± SD from three independent experiments performed in triplicate. *Indicates significant difference as compared to the untreated control group (P < 0.05). **Indicates significant difference from the β-elemene treated alone (P < 0.05).

Studies demonstrated the potential links of DNMT1 and EZH2 that trigged the cellular responses in different cancer cell types^35, 39–41^. Thus, we next elucidate the potential interaction between DNMT1 and EZH2 that may be involved in the overall responses of β-elemene. As shown in Fig. 4A,B, exogenously expression of DNMT1 significantly reversed the effect of β-elemene on EZH2 protein levels, while no effect was observed in *vice versa*. As expected, exogenously expressed DNMT1 also overcame the effect of β-elemene on EZH2 promoter activity (Fig. 4C). On the contrary, silencing of DNMT1 by siRNA approaches further reduced EZH2 protein expression (Fig. 4D). These results suggested that DNMT1, acting as an upstream molecule, not only regulated but also interacted with EZH2, thereby contributing to the overall effects of β-elemene on inhibition of NPC cell growth.

### Exogenously expression of DNMT1 and EZH2 feedback reversed the effect of β-elemene on phosphorylation of Stat3 and cell growth inhibition

**Figure 5:**
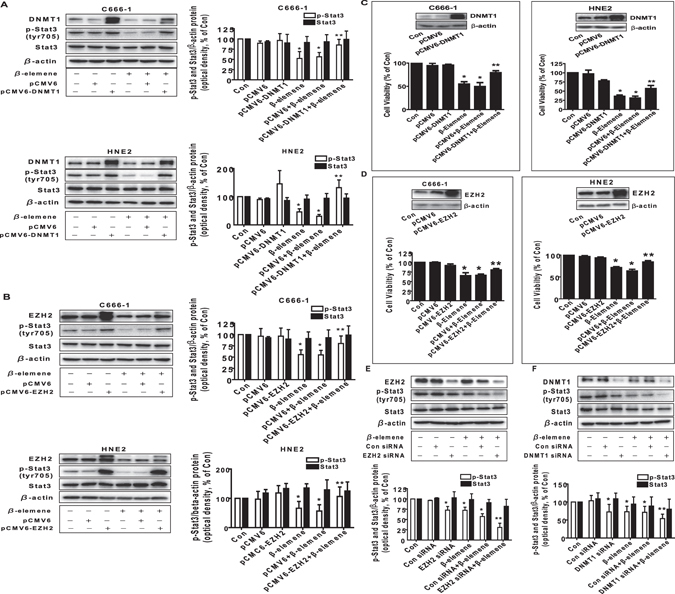
Exogenously expression of DNMT1 or EZH2 feedback reversed the effect of β-elemene on phosphorylation of Stat3 and cell growth inhibition. (**A,B**) C666-1 and HNE2 cells were transfected with control or DNMT1 or EZH2 expression vectors for 24 h prior to exposure of the cells to β-elemene (20 μg/mL) for an additional 24 h. Afterwards, Western blot analysis were used measure the levels of DNMT1 and EZH2, and p-Stat3, Stat3 proteins using corresponding antibodies. (**C,D**) C666-1 and HNE2 cells were transfected with control or DNMT1 or EZH2 expression vectors for 24 h prior to exposure of the cells to β-elemene (20 μg/mL) for an additional 48 h. Afterwards, cell proliferation was examined by MTT assays as described in the Materials and Methods section. Insert blots represented protein expressions of DNMT1 and EZH2. (**E,F**) C666-1 cells were transfected with control or DNMT1 or EZH2 siRNAs for 30 h prior to exposure of the cells to β-elemene (20 μg/mL) for an additional 24 h. Afterwards, Western blot analysis were used measure the levels of DNMT1 and EZH2, and p-Stat3, Stat3 protein using corresponding antibodies. The figures are representative cropped gels/blots that have been run under the same experimental conditions. Values in bar graphs were given as the mean ± SD from three independent experiments performed in triplicate. *Indicates significant difference as compared to the untreated control group (P < 0.05). **Indicates significant difference from the β-elemene treated alone (P < 0.05).

In order to further determine the role of DNMT1 and EZH2, we have transfected the plasmids expressing high levels of DNMT1 and EZH2 genes. As shown in Fig. 5A and B, exogenously expression of DNMT1 or EZH2 positive feedback reversed the effect of β-elemene on phosphorylation of Stat3 (Fig. 5A,B). Interestingly, they also overcame the β-elemene-inhibited cell growth in C666-1 and HNE2 cells (Fig. 5C,D). These findings implied that the possible parallel regulatory pathways were also existed involving in the DNMT1- and EZH2-mediated the effects of β-elemene on cell growth inhibition. Of note, overexpression of EZH2 and DNMT1 had no effect on total Stat3 protein expression (Fig. 5A,B). Intriguingly, silencing of EZH2 and DNMT1 by siRNA approaches found to feedback strengthen the effect of β-elemene on phosphorylation of Stat3 (Fig. 5E,F). Together, the findings above suggested that positive regulatory loops between the phosphorylation of Stat3 and EZH2, DNMT1 were existed in this process.

### *In vivo* anti-tumor efficacy of β-elemene in subcutaneous NPC tumor-bearing nude mice model

**Figure 6:**
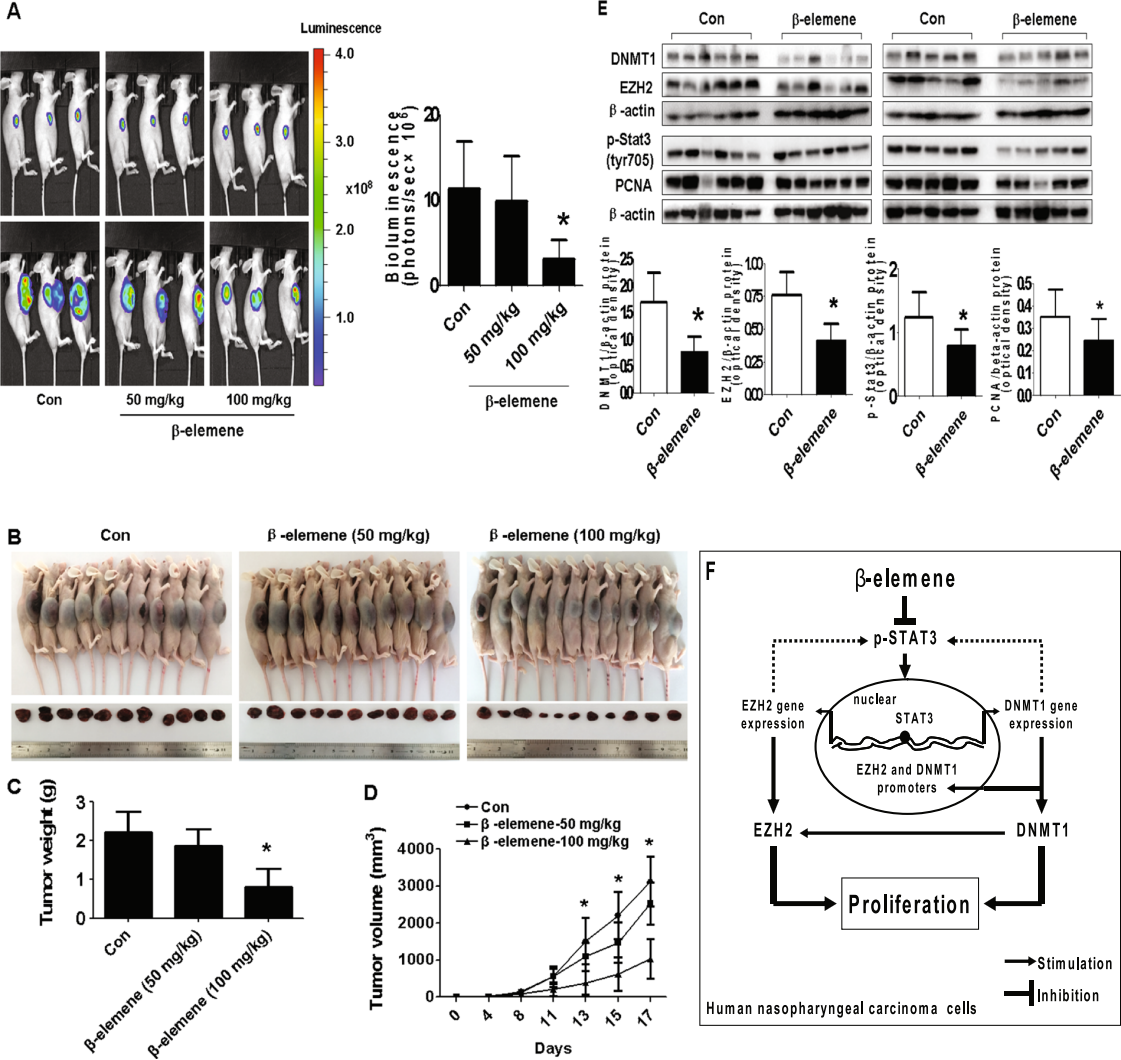
*In vivo* anti-tumor efficacy of β-elemene in subcutaneous lung tumor-bearing nude mice model. Mice (n = 11/group) were divided to 3 groups [Con (saline), Low (L, 50 mg/kg) and High (H, 100 mg/kg)], and β-elemene was given at the 10^th^ day after tumor cells injection by gavages for up to 17 days. (**A**) The xenografts were assessed by *in vivo* bioluminescence imaging at the day 1 and the end of the experiments (on day 17). The tumor growth was monitored by injecting luciferin in the mice followed by measuring bioluminescence using IVIS Imaging System. Imaging and quantification of signals were controlled by the acquisition and analysis software living image as described in the Materials and Methods section. Representative images are shown. (**B**) The photographs of β-elemene or vehicle-treated xenografts derived from nude mice are shown. (**C,D**) The xenografts were harvested on day 17, and the volume and weight of tumors were measured. (**E**) At the end of the experiments, xenografted tumors from the high dose and control groups were isolated from individual animals and the corresponding lysates were processed for detecting DNMT1, EZH2, p-Stat3, and PCNA by Western blot. β-actin was used as loading control. The bar graphs represented the tumor weight and volume of mice results of as mean ± SD. *Indicates the significant difference from the untreated control (p < 0.05). (**F**) The diagram shows that β-elemene inhibits NPC growth through inactivation of Stat3, followed by reduction of DNMT1 and EZH2 expression. The reciprocal communication between DNMT1 and EZH2, the mutual regulatory loops of Stat3 with DNMT1 and EZH2 contribute to the overall responses of β-elemene in this process.

We also tested the effect of β-elemene in NPC tumor growth and DNMT1, EZH2 expression in nude mouse xenografted cancer model. The therapeutic efficacy of β-elemene on the growth of C666-1 cells was assessed *in vivo*. Luciferase-expressing C666-1 cells were injected subcutaneously into nude mice. Mice bearing xenografted tumors were treated by gavages daily for different doses of β-elemene (50 and 100 mg/kg), which based on other studies^7, 42^, for up to 17 days. We found that, compared to the control group, the high dose of β-elemene-treated mice group showed a significant suppression of tumor growth as assessed by the Xenogen IVIS200 System (Fig. 6A). In addition, we noticed a significant reduction of the tumor weight and volume in the high dose of β-elemene-treated mice group as compared to that in the control one (Fig. 6B–D). By Western blot analysis, fresh tumors harvested from the aforementioned experiments showed that β-elemene at high dose treatment significantly inhibited DNMT1 and EZH2 protein expressions and phosphorylation of Stat3 as compared to that in the control group (Fig. 6E). As expected, a decreased protein expression of proliferating cell nuclear antigen (PCNA), the marker of cell growth, was also observed in the high dose β-elemene treated group as compared to that in the control one (Fig. 6E).

## Discussion

β-elemene is a broad-spectrum anticancer drug extracted from the traditional Chinese medicinal herb Curcuma wenyujin. Growing evidence suggests that β-elemene has been shown to be effective against a wide variety of tumors^7–9, 17, 43^. However, there was no published data demonstrating the association and potential of β-elemene in controlling of NPC. In the present study, we for the first time showed that β-elemene inhibited growth of NPC cells, suggesting a new therapeutic strategy for the treatment of this malignancy.

A large body of evidence demonstrated the significant role of multiple signaling pathways and potential targets (genes or/and proteins) involved in the anti-tumor effects of β-elemene in various cancer types^7, 17, 43, 44^. However, the detailed molecular mechanism underlying this remains largely to be elucidated. More importantly, no solid evidence has shown the effect of β-elemene on influencing NPC cells. Therefore, the potential singling pathways and targets involved in the anti-NPC effects by β-elemene remained unknown. Herein, we found that inhibition of Stat3 signaling pathway was involved in the inhibitory responses of the β-elemene in human NPC cells. Activation of Stat3 regulates the expression of numerous genes associated with tumor invasion, metastasis and survival^45^. On the contrary, blockade of Stat3 function is sufficient to inhibit tumor cell growth and induce apoptosis^46^. Thus, inhibition of Stat3 holds great promise for future cancer treatment concepts. As evidence by our findings that exogenously expressed Stat3 overcame the effect of β-elemene on NPC cell proliferation implied the important role of this transcription factor inactivation in mediating the anti-NPC responses. Overexpression of Stat3 has been shown to resist the anti-cancer effects, resulting in poor prognosis in several malignancies^30, 47–51^. Thus, Stat3 not only provided a potential mechanism of action for tyrosine kinase inhibitors but also represented new molecular targets for novel NPC therapy. However, recent study also indicated that Stat3 could play tumor-suppressive role in K-ras mutant lung cancer as K-ras mutant lung tumors had reduced Stat3 levels^52^. Therefore, the true functions of this transcription factor need to be elucidated further.

We demonstrated that the inhibition of DNMT1 and EZH2 mediated the effect of β-elemene on NPC cell growth. As critical epigenetic factors and tumor promoters, increased expressions of DNMT1, one of the DNA methyltransferases, and EZH2, one of the polycomb proteins^39, 53^, were found in several different cancer cells including NPC one and inhibited tumor suppressor genes^18, 27, 38, 54^. In addition, overexpressions of DNMT1 and EZH2 were associated with induced proliferation and aggressive metastasis behavior, resulting in poor prognosis in many cancer types. Thus, targeting of DNMT1 and EZH2 may be of therapeutic benefits for patients with certain malignancies^55–57^. Our results showed that β-elemene-inhibited expressions of DNMT1 and EZH2 were through suppressing Stat3 signaling suggesting that Stat3 was an upstream molecule of these two oncogenetic factors. Consistent with this, previous studies found that the DNMT1 and EZH2 gene promoters contained Stat3 DNA binding sites and that regulation of Stat3 influenced the expression of DNMT1 and EZH2, and even its downstream signaling^36, 37^. One early study observed that viral latent membrane protein 2A (LMP2A) increased DNMT1 expression, and that knockdown of STAT3 by siRNA counteracted LMP2A-mediated DNMT1 expression in gastric carcinoma cells^58^. Nevertheless, the true role of Stat3 activation and expression in mediating the anti-NPC effect by β-elemene still remained to be determined.

The association between DNMT1 and EZH2 has been reported^35, 39–41^. EZH2 may influence DNA methylation by direct interacting with DNA methyltransferases^41^. EZH2 was found to bind and recruit DNMT1 to modulate DNA methylation^39^, and could directly affect the function of DNMT1^40^. Study also demonstrated an interaction between DNMT1 and EZH2 in gastric cancer and glioma cells^35^. In contrast, our results implied that DNMT1 may be an upstream signal of EZH2, stimulating expression of EZH2 via transcriptional and translational levels, and that the correlation between DNMT1 and EZH2 contributed to the overall responses of β-elemene in inhibition of NPC cell proliferation. In line with this, our previous studies and others have demonstrated the upstream role of DNMT1 on EZH2. Exogenously expression of DNMT1 antagonized polyphyllin I (PPI), a bioactive phytochemical extracted from the Rhizoma of Paris polyphylla, and ursolic acid, a pentacyclic triterpenoid, in reduction of EZH2 expression in human lung cancer cells^59, 60^. Moreover, one recent study have showed that knockdown of EZH2 by siRNA was not associated with any significant alteration of DNMT1, indicating that EZH2 expression was regulated by DNMT1 but not the reverse, in glioblastoma cells^33^. Thus, more studies are required to elucidate this interplay axis. Intriguingly, we also demonstrated the feedback regulatory loops of EZH2 and DNMT1 on phosphorylation of Stat3 signaling, suggesting the oncogenic role and regulatory circuit of these transcriptional factors in mediating the overall responses of β-elemene on controlling of NPC cell growth. There was less information available for these mutual feedback regulatory axis; one study showed that regulation of EZH2 was involved in positive feedback loop with β-catenin/transcription factor 4 (TCF4) and Stat3 signaling in glioblastoma cells^61^. And another report demonstrated that activated interleukin-6/Stat3 signaling could induce suppressor of cytokine signaling 3 (SOCS3) methylation via DNMT1, resulting in pancreatic cancer growth and metastasis^62^. Evidence showed that EZH2 could bind to Stat3, and the EZH2-Stat3 interaction led to enhance Stat3 activity in glioblastoma cells. Therefore, inhibition of EZH2 attenuated Stat3 activity^63^. Moreover, the anti-cancer effects of β-elemene have also been observed through affecting other signaling pathways, such as Notch1, phosphatidylinositol 3-kinase (PI3K)/protein kinas B (Akt) and mitogen-activated protein kinase (MAPK), among others^17, 64, 65^. And some of which may also regulate and interact with Stat3 signaling through feedback regulation loops in different cell types^65, 66^. However, whether this occurs in our system requires being determined. Nevertheless, the true insight into the mechanisms underlying the feedback regulations of Stat3 by EZH2 or/and DNMT1, such as potential intermediates or other kinase signaling pathways being involved, expected to be more complicated than we thought, and required to be determined with more experimental approaches in the future. Moreover, we demonstrated that both EZH2 and DNMT1 were directly involved in the β-elemene-inhibited cell proliferation; this, together with the results from the feedback regulatory loops, implied that other parallel pathways that converged on the effects of β-elemene could be existed in this study. This further indicated that more experiments are required to elucidate this in the future.

More importantly, our *in vivo* data were consistent with the findings from that *in vitro*, confirming the effect of β-elemene on NPC cell growth inhibition, regulation of STAT3a signaling, and EZH2, DNMT1 expression. The given doses of β-elemene were similar with others demonstrating the significant effects on the inhibition of other cancer cell types^7, 8, 42^. More importantly, this was showed to be comparable to that putative therapeutic one achieved in several other studies summarized in one systematic review^67^. We believed that more studies are needed to elucidate the involvement of these two markers played in the anti-NPC property using cells stable transfected with exogenously expression vectors containing DNA code regions (exons) of EZH2 and DNMT1 genes in nude mice model, respectively.

In summary, our results show that β-elemene inhibits NPC growth through inactivation of Stat3, subsequently reducing DNMT1 and EZH2 expressions. The reciprocal inter-regulations between DNMT1 and EZH2, and the mutual regulatory loops of Stat3 with DNMT1 and EZH2 contribute to the overall responses of β-elemene in this process (Fig. 6F). This study uncovers a novel mechanism with complex mutual regulatory networks by which β-elemene inhibits NPC cell growth and emphasizes critical roles for DNMT1 and EZH2 in the growth and progression of NPC, and highlights the potential for optimizing therapeutic strategy of NPC.

## Materials and Methods

### Reagents and cell culture

The antibodies against DNMT1 and EZH2 were obtained from Cell Signaling Technology, Inc. (Beverly, MA, USA). The antibodies against phosphor-and total Stat3 were purchased from Merck Millipore (Billerica, MA, USA). MTT powder was purchased from Sigma Aldrich (St. Louis, MO, USA). Lipofectamine 3000 reagent was purchased from Invitogen (Carlsbad, CA, USA). β-elemene was purchased from Chengdu Must Bio-technology Company (Chengdu, Sichuan, China). The drugs were freshly diluted to the final concentration with culture medium before experiment. Human NPC cells C666-1 and HNE2 were obtained from the Cell Line Bank at the Laboratory Animal Center of Sun Yat-sen University (Guangzhou, China) and the Chinese Academy of Sciences Cell Bank of Type Culture Collection (Shanghai, China). All cell lines have been tested and authenticated for absence of *Mycoplasma*, genotypes, drug response, and morphology. C666-1 is commonly studied Epstein-Barr virus (EBV)-positive and undifferentiated NPC cells. HNE2 is EBV-negative and poorly differentiated NPC cell line. The sensitivity of HNE2 cells to therapies was higher than that of C666-1 cells. However, there are scarce information available for the short tandem repeat loci in NPC cell lines^68^ although studies to map chromosomal loci linked to susceptibility genes predisposing for NPC families were reported^69, 70^. The cells were cultured at 37 °C in a humidified atmosphere containing 5% CO_2_. The culture medium consisted of RPMI 1640 medium (Life Technologies, Carlsbad, CA, USA) supplemented with 10% (v/v) heat-inactivated fetal bovine serum (Thermo Fisher Scientific Inc, Waltham, MA, USA), 100 µg/mL streptomycin and 100 U/mL penicillin. When cells reached 70% confluence, they were digested with 0.25% trypsin for passage for the following experiments.

### Cell viability assay

The cells viability experiments were performed by 3-(4,5-dimethylthiazol-2-yl)-2, 5-diphenyltetrazolium bromide (MTT) assays as described previously^17, 71^. NPC cells were harvested, counted and seeded into a 96-well μL (2 × 10^4^ cells/well). The cells were treated with increasing concentrations of β-elemene for up to 72 h. Afterwards, MTT solution (10 μL/well, 5 g/L) mixed with NSCLC cells were incubated at 37 °C for an additional 4 h. After removing the supernatant, 150 µL dimethyl sulfoxide solutions were added into each well and oscillated for 10 min. The fluorescence signal was read through the use of ELISA reader (Perkin Elmer, Victor X5, Waltham, MA, USA). Cell viability (%) was calculated as follows: (absorbance of test sample/absorbance of control) × 100%.

### Cell cycle analysis

This procedure was reported previously^17^. Briefly, NPC cells were cultured in 6-well plastic plates at 2 × 10^5^ cells/well and treated with increased doses of β-elemene for 24 h. Afterwards, the cells were harvested, washed with phosphate-buffered saline (PBS), and resuspended in 500 μL of cold PBS and ethanol (1.5 mL) for 2 h at 4 °C. Afterwards, the fixed cells were incubated in 1 mL of 0.1% sodium citrate containing propidium iodide (PI) and RNase for 30 min at room temperature. The cells were washed and subjected to FACS Calibur flow cytometric analysis (FC500, Beckman Coulter, FL, USA), and the proportion of cells within the G0/G1, S, and G2/M phases of the cell cycle were analyzed using the MultiCycle AV DNA Analysis software (Phoenix Flow Systems, Inc. San Diego, CA, USA).

### Western blot analysis

The Western blot procedures were performed as reported previously^17, 71^. Briefly, after determining the protein concentrations by the Bio-Rad protein assay. The cell lysates containing equal concentration of protein were solubilized in SDS-sample buffer and separated on SDS polyacrylamide gels. Membranes (Millipore, Billerica, MA, USA) were incubated with antibodies against phosphor- and total Stat3, DNMT1 and EZH2 (1:1000 and 1:2000, respectively). The membranes were washed and incubated with a secondary goat antibody raised against rabbit IgG conjugated to horseradish peroxidase, and transferred to freshly made ECL solution (Immobilon Western; Billerica, MA, USA), followed by observing the signals under the Molecular Imager ChemiDoc XRS Gel Imagine System (BioRad, Hercules, CA, USA) and documenting the results.

### Treatment with EZH2 and DNMT1 siRNAs

The detailed procedure was reported previously^71^. In brief, cells were seeded in 6-well or 96-well culture plates in RPMI 1640 medium containing 10% FBS (no antibodies), grown to 60% confluence, and the siRNAs (up to 50 nM) of EZH2 (obtained from Cell Signaling Technology, Inc.), DNMT1 and control (purchased from Santa Cruz Biotechnology, Inc, Dallas, Texas, USA) were transfected using Lipofectamine 3000 according to the manufacture’s protocol, and incubated for up to 24 h. Afterwards, the cells were harvested and resuspended in the presence of β-elemene for the indicated time for all other experiments.

### Transient transfection assays

The procedure was reported previously^72^. In brief, NPC cells were seeded at a density of 5 × 10^5^ cells/well in 6-well dishes and grown to 50–60% confluence. For each well, 2 µg of the control (pCMV6) and expression constructs containing Myc-DDK-tagged- or Myc/FLAG-tagged ORFs of human Stat3, DNMT1 or EZH2 obtained from OriGene Technologies, Inc. (Rockville, MD, USA), at a final concentration of 2–3 μg/mL were transfected into the cells with the Lipofectamine 3000 reagent. Cells were incubated for 24 h at 37 °C, then treated with β-elemene for the indicated time for all other experiments. In separated experiment, cell were transfected with pEZX-PG04-EZH2 promoter construct linked Gaussia luciferase (GLuc) gene and secreted alkaline phosphatase (SEAP) internal control obtained from GeneCopoeia, Inc. (GeneCopoeia, Inc., Rockville, MD, USA). The preparation of cell extracts and measurement of luciferase activities were determined using the Secrete-Pair Dual Luminescence Assay Kit (GeneCopoeia, Inc., Rockville, MD, USA). Gaussia luciferase activity was normalized with SEAP within each sample.

### Xenograft experiments

Animal experiments were performed in accordance with the Guide for Care and Use of Laboratory Animals and all protocols were approved by Institutional Animal Care and Use Committee Animal Care of Guangdong Provincial Hospital of Chinese Medicine. A total of 33 eight-week-old female nude mice were obtained from Guangdong Provincial Research Center for Laboratory Animal Medicine (Foshan, Guangdong, China) and maintained at the Animal Center in our Hospital in a specific pathogen-free environment with food and water provided. C666-1 cells carrying luciferase report gene (C666-1-Luc, obtained from the Guangzhou Land Biological Technology Co., Guangzhou, China) (1 × 10^6^ cells) in 100 μL PBS were injected subcutaneously in the dorsal side of nude mice. Xenografts were allowed to grow for over one week when the initial measurement was made with calipers and with bioluminescence imaging (BLI) using the IVIS-200 Imaging System (Xenogen Corporation, Berkeley, CA). Briefly, the mice were randomly divided into control, low (50 mg/kg), and high doses (100 mg/kg) of β-elemene via gavages for up to 17 days (n = 11/group)^7, 42^. For BLI procedure, at the beginning and the end of treatment, mice were anesthetized by inhalation of 2% isoflurane. Each set of mice were injected intraperitoneally with 150 mg/kg D-luciferin (Xenogen; PerkinElmer, Waltham, MA, USA) in approximately 200 μL. The intensity of BLS in the luminescent area of the tumor was determined by Living Image 3D software (version 1; Xenogen). Tumor volume measurements were calculated using the formula for an oblong sphere: volume = (width^2^ × length). Luciferase signal is reported as photons/sec. The body weights of the mice were measured once a week. All mice were sacrificed on 17 days after each treatment using CO_2_ for euthanasia. The corresponding xenografted tumors were isolated and processed for detecting the phosphorylation of Stat3, DNMT1 and EZH2 proteins, proliferation marker PCNA by Western blot.

### Statistical analysis

All experiments were repeated a minimum of three times. Data are represented as mean ± SD, unless otherwise indicated. Differences between groups were assessed by one-way ANOVA and significance of difference between particular treatment groups was analyzed using GraphPadPrism5.0 software (LaJolla, CA, USA). The results in graphs were presented relative to the control. Differences with a probability of 95% (*p* < 0.05) were considered to be significant.
